# Guidance for Home Enzyme Replacement Therapy in Children and Adolescents Diagnosed with Mucopolysaccharidoses: A Scoping Review Protocol

**DOI:** 10.3390/ijerph22111686

**Published:** 2025-11-07

**Authors:** Vinícius Rodrigues de Oliveira, Yarytizza Nycoly Fernandes Martins, Juliana Iscarlaty Freire de Araújo, Eunice Fernandes da Silva, Margarita Baeza Fuentes, Jonas Sâmi Albuquerque de Oliveira

**Affiliations:** 1 Departament of Nursing, Federal University of Rio Grande do Norte, Natal 59078-970, Brazil; yarytizzaf@hotmail.com (Y.N.F.M.); jonas.albuquerque@ufrn.br (J.S.A.d.O.); 2 Departament of Public Health, Federal University of Rio Grande do Norte, Natal 59078-970, Brazil; jiscarlaty@gmail.com; 3 Pediatric Outpatient Clinic, Onofre Lopes University Hospital, Natal 59010-054, Brazil; lucasanneluna@gmail.com; 4 Departament of Nursing, University of Santiago de Chile, Santiago 9170022, Chile; margarita.baeza@usach.cl

**Keywords:** mucopolysaccharidosis, enzyme replacement therapy, therapy, home infusion, child, adolescent

## Abstract

Enzyme replacement therapy is one of the main alternatives in the treatment of mucopolysaccharidosis. Conventionally, this therapy is administered in an outpatient setting, but this can be carried out at home, where the patient feels most comfortable. However, there is still a need for guidance on how to implement the use of this therapy in the home setting. This study aims to present the protocol of a scoping review that aims to identify and map the main existing guidance for the implementation of home-based enzyme replacement therapy in children and adolescents with mucopolysaccharidosis. The review protocol was developed based on the methodological guidance proposed by Peters and the PRISMA-P checklist and was registered on the Open Science Framework platform, available at OSF.IO/QUSB6. The searches will be carried out in the following databases: CINAHL, Cochrane Library, Embase, LILACS, MEDLINE, Science Direct, Scopus, and Web of Science. Gray literature will be searched in the CAPES/Brazilian Theses and Dissertations Catalog, the Brazilian Ministry of Health website, Google Scholar, the National Institute for Health and Care Excellence, and the WHO IRIS. Articles will be selected according to predefined eligibility criteria and extracted using a standardized collection form. The results of this review are expected to provide valuable insights for researchers regarding the main guidance for the implementation of home-based ERT in children and adolescents with mucopolysaccharidosis.

## 1. Introduction

Mucopolysaccharidoses (MPSs) are a subset of rare diseases resulting from the incomplete degradation of glycosaminoglycans (GAGs) in the body, leading to progressive respiratory, cardiovascular, musculoskeletal, auditory, and visual impairment [1]. In the global epidemiological context, it is estimated that the combined prevalence of MPS is between 0.036 and 16.9 per 100,000 live births, with higher rates reported in countries such as Saudi Arabia, Portugal, Brazil, the Netherlands, and Australia [2].

Given this reality, diagnosis during childhood and adolescence, with an emphasis on the early recognition of signs and symptoms, is essential for preventing more-severe clinical impairments and promoting patient well-being [3].

Regarding therapeutic possibilities for the treatment of MPS, enzyme replacement therapy (ERT) is considered to be one of the main approaches. This treatment consists of the infusion of enzymes that act to minimize enzymatic errors resulting from metabolic alterations, and it is administered periodically via intravenous infusion [4].

ERT was first implemented for Gaucher disease during the 1990s. Subsequently, in 2003, this therapy began to be used for the treatment of mucopolysaccharidosis type I (Hurler syndrome), and in the years 2005, 2006, and 2014, MPS types VI, II, and IV were included in this therapeutic approach, respectively. This modality can be administered either in a hospital setting or at the patient’s home, depending on a risk/benefit assessment undertaken by the physician, the patient, and the nurse responsible for the patient’s care [5].

Home administration of ERT stands out as one of the best alternatives, as, when compared to hospital-based therapy, it is perceived as less stressful and more comfortable. Aiming to improve adherence to intravenous therapies, the experience of home-based ERT gained strength during the global pandemic, demonstrating that home infusions enhance quality of life by promoting patient autonomy and independence, as well as improving disease management [6].

From an international perspective, countries such as the United Kingdom, the United States, Italy, and the Netherlands have already consolidated and recommended home-based ERT, primarily due to greater patient adherence, in addition to demonstrating safety and efficacy [7]. However, in Brazil and other developing countries, there is still limited guidance on the implementation of home-based ERT for patients with MPS, highlighting a significant gap in the literature and care practices.

In light of this scenario, there is a need to develop studies that provide theoretical and practical support for the implementation of home-based ERT in children and adolescents diagnosed with MPS. This initiative aims to improve patients’ quality of life, increase treatment adherence, and promote the principles of comprehensiveness and universality in healthcare.

Accordingly, the present study aims to present the protocol for a scoping review that seeks to identify and map the main existing guidance for carrying out home enzyme replacement therapy in children and adolescents diagnosed with MPS.

## 2. Materials and Methods

This protocol was developed based on the methodological guidance of Peters et al. (2022) [8] and the PRISMA-P checklist [9] (available in supplementary materials). The registration was performed on the Open Science Framework (OSF) platform and is available for consultation at OSF.IO/QUSB6. This publication aims to ensure transparency in the review development process and to serve as a reference for other researchers interested in conducting similar studies.

The conduct and reporting of the scoping review will follow the recommendations established by the JBI Manual for Evidence Synthesis (2024 Edition) [10] and the PRISMA-ScR checklist [11], respectively.

### 2.1. Research Question

The PCC framework was adopted to develop the research question, where P (population)—children and adolescents diagnosed with MPS; C (concept)—guidance for enzyme replacement therapy; and C (context)—home setting.

Thus, the following research question was formulated: What is the main guidance for administering home-based enzyme replacement therapy to children and adolescents with mucopolysaccharidosis?

### 2.2. Eligibility Criteria

To be considered eligible, each study must meet the criteria previously established by the authors, based on the PCC framework, as described below (Table 1).

Eligible sources of evidence will include primary and secondary studies, official documents (guidelines, manuals, and protocols), dissertations, theses, books, and publications from scientific conference proceedings, as long as they directly address the proposed research question. No restrictions will be applied regarding the year of publication or language, in order to ensure a comprehensive and representative overview of the scientific literature on the topic.

For materials published in languages other than Portuguese or Spanish, a translation plan will be implemented using automated translation tools. When necessary, and to ensure the accuracy of the information, support from specialized academic translation services provided by the authors’ affiliated institutions will be sought. Evidence not available in full text and reflective studies will be excluded, as they do not meet the methodological standards established for this review.

### 2.3. Data Source and Search Strategy

Ten international data sources will be selected, considering the broad scope of the researched topic. The search for the conventional literature will be conducted through the following sources: Cumulative Index to Nursing and Allied Health Literature (CINAHL via EBSCO), Cochrane Library, Embase, Latin American and Caribbean Health Sciences Literature (LILACS), Medical Literature Analysis and Retrieval System Online (Medline via PubMed), Science Direct, Scopus, and Web of Science. The search for gray literature will be conducted through the CAPES Foundation’s Catalog of Theses and Dissertations (Brazil), Google Scholar, the Brazilian Ministry of Health website, the National Institute for Health and Care Excellence (NICE), and the World Health Organization Institutional Repository for Information Sharing (WHO IRIS).

To formulate a highly sensitive search strategy tailored to each data source, the following steps were taken: (I) consulting Health Sciences Descriptors (DeCS), Medical Subject Headings (MeSH), and Emtree; (II) identifying alternative terms/uncontrolled descriptors; (III) organizing descriptors and alternative terms into a table using Microsoft Office Word, 2016 version; and (IV) developing the search strategy, associating terms with Boolean operators: AND, OR, or NOT.

It is emphasized that the search strategies were previously reviewed by the authors, taking into account the specificities of each data source, until the ideal search key was reached in light of the scoping review’s objective.

The use of the Boolean operator “NOT” proved to be necessary, as its absence in the search strategy resulted in the retrieval of a substantial number of studies related to other conditions, such as Gaucher disease, Fabry disease, and neoplasms, with little or no relevance to MPS, as these conditions present distinct clinical characteristics. The predominance of these irrelevant records significantly compromised the specificity of the search and negatively impacted the initial screening process by substantially increasing the volume of the literature not aligned with the research question. The Table 2 illustrates the defined strategies:

### 2.4. Selection of Evidence

Upon completion of the database search, all retrieved references will be exported to Zotero software version 7.0.21 (George Mason University, Fairfax, VA, USA) to optimize bibliographic management and enable the identification and removal of duplicate records. After this initial screening, the remaining references will be imported into Rayyan software (2024 version, Qatar Computing Research Institute, Doha, Qatar), where the study selection will be carried out independently by two reviewers based on the previously established eligibility criteria.

The records will be screened by two reviewers independently, who will be blinded to each other, to avoid any influence on the selection of material. In cases of conflict regarding the inclusion or exclusion of a study, the final decision will be made by a third reviewer: a nurse with a Ph.D. in nursing and experience in caring for children and adolescents with MPS.

To ensure the reliability of assessments and minimize bias in the study selection process, a pilot test will be conducted in which inter-reviewer agreement will be evaluated using the Kappa coefficient. The team must achieve a minimum agreement level of 80%. If this threshold is not met, additional training will be provided, including a joint review of the eligibility criteria. It is important to note that the study selection process will only begin after the minimum required level of agreement has been reached.

The results of the evidence selection process will be presented in the final version of the scoping review, accompanied by a flowchart designed according to PRISMA-ScR guidelines.

### 2.5. Data Extraction

Data extraction will be performed independently by two researchers using the Google Forms platform, through a customized form specifically designed to meet the objectives of this review.

The choice of this tool is justified by its functionality in systematizing and organizing the collected information, enabling the automated generation of spreadsheets and charts. These resources will facilitate the descriptive analysis of the extracted data, contributing to a clear and structured presentation of the results in the final version of the review.

The form template can be accessed through the following link: https://forms.gle/miYVswm5DRXDN7K39. It is important to note that this is a preliminary version of the extraction instrument and may be modified if the researchers identify the need for changes. If any modifications are made, they will be detailed in the final scoping review report.

The form is organized into two sections: The first section concerns the extraction of data related to the bibliometric profile of the sample studies, collecting information such as publication year, authors, country of research, objective, type of document, and type of study.

The second section addresses the content related to the guidance for home-administered enzyme replacement therapy in children and adolescents with MPS, where the following data will be extracted: type of MPS diagnosed in the patient; number of patients who received home-based ERT in the study; general guidance for home-based ERT; nursing guidance; minimum number of months of outpatient or hospital-based ERT before starting home-based ERT; medications used in the infusion; minimum age at which patients started home-based ERT; adverse events during the procedure and their management; and ages of the patients who underwent home-based ERT.

Nursing care guidance is included because the presence of a nurse is required in both outpatient and home-based ERT settings.

### 2.6. Data Analysis

The data will be analyzed quantitatively through the presentation of graphs and percentages describing the bibliometric profile of the studies included in the sample. For qualitative synthesis, the PAGER framework will be employed. This strategy represents a systematic approach developed to enhance the analysis and reporting of scoping reviews.

The PAGER framework is a mnemonic that stands for five interrelated analytical domains: patterns, advances, gaps, evidence for practice, and research recommendations. This approach enables the clear and accessible mapping of the main thematic findings, identifies advancements in the studied area, highlights deficiencies in the existing literature, and emphasizes practical implications and future research directions [12].

The adoption of the PAGER framework was motivated by the need for a more structured analytical approach that facilitates the organization and practical application of knowledge. This framework enables the identification of patterns and advances in the literature, the mapping of knowledge gaps, and the development of recommendations that are relevant both to clinical practice and future research. Its use is particularly appropriate in reviews with direct implications for healthcare delivery, such as the administration of ERT at home for individuals with MPS. By employing the PAGER structure, it becomes possible to translate research findings into concrete guidance for clinical decision-making and the development of more effective care policies and protocols.

The Table 3 describes each element of the PAGER acronym and the type of findings that it is intended to capture.

#### Methodological Quality Assessment

The levels of evidence of the included studies will be determined using the JBI Levels of Evidence. Considering the potential inclusion of guidelines and protocols in the final review sample, the JBI Grades of Recommendation will also be applied to support the classification of these recommendations based on the strength of the available evidence.

## 3. Discussion

In recent years, it has become possible to conduct more robust clinical trials on enzyme replacement therapy (ERT), and these studies have reaffirmed the effectiveness of this therapy for patients diagnosed with mucopolysaccharidosis (MPS), while also expanding the scope of information regarding adverse reactions and their management. This progress has enabled the recommendation of more specific approaches for the administration of ERT, including home-based therapy [6].

Despite this advancement in knowledge, home-based ERT is still not a reality in many countries, and its implementation faces several challenges, particularly regarding guidance on how to conduct this process [13]. Moreover, in low- and middle-income countries, there is limited interest in transitioning care to the home setting. This gap may be partially attributed to the low prioritization of rare diseases in comparison to more prevalent health conditions.

Although ERT is already established as one of the main therapeutic alternatives for patients with MPS, as previously discussed, its administration in the home setting presents new challenges. Among these is the need for further scientific investigation to provide theoretical and practical support for guiding the implementation of this therapeutic modality [14].

Given this scenario, the available literature includes a range of documents addressing home-based ERT from various perspectives, including case reports, expert consensus statements, and national and international guidelines. However, these materials differ in their format, scope, and level of detail, which hinders an integrated view of the current recommendations available to support clinical practice.

Therefore, it becomes essential to develop a study with a methodological approach capable of mapping and organizing this heterogeneous body of evidence, identifying clinical elements and gaps in knowledge. The proposed scoping review assumes this role by allowing for a comprehensive synthesis of the existing guidance related to the home administration of ERT in children and adolescents with MPS.

## 4. Conclusions

The findings of this scoping review are expected to contribute to expanding the clinical understanding of the topic, supporting the development of tools and strategies aimed at implementing this therapeutic modality, and identifying key gaps for future research. By fulfilling these objectives, the resulting study will align with the core principles of scoping reviews, providing a conceptual foundation to inform decisions in both research and clinical practice.

## Figures and Tables

**Table 1:** Eligibility criteria according to the PCC framework.

PCC	Inclusion Criteria	Exclusion Criteria
Population	Individuals diagnosed with mucopolysaccharidosis who are between the ages of 0 and 19 years (the age range in which the World Health Organization [WHO] considers the individual to be a child or adolescent).	Individuals outside the age limits of childhood or adolescence established by the WHO.
Concept	Guidance for home enzyme replacement therapy.	Guidance for home enzyme replacement therapy carried out to treat diseases other than mucopolysaccharidosis.
Context	Home environment.	Hospital or outpatient setting.

**Table 2:** Search strategy according to the data sources.

Conventional Literature		
Data Source	Search Strategy	Preliminary Results
Cochrane Library	Child OR Children in Title Abstract Keyword OR Adolescence OR Adolescents OR ‘Adolescents, Female’ OR ‘Adolescent, Female’ OR ‘Adolescent, Male’ OR ‘Male Adolescents’ OR Youth OR Youths OR Teen OR Teens OR Teenager OR Teenagers in Title Abstract Keyword AND mucopolysaccharidoses OR mucopolysaccharidosis in Title Abstract Keyword AND ‘Enzyme Replacement Therapy’ OR ‘Enzyme Replacement Therapies’ OR ‘Replacement Therapies, Enzyme’ OR ‘Replacement Therapy, Enzyme’ OR ‘Therapies, Enzyme Replacement’ OR ‘Therapy, Enzyme Replacement’ in Title Abstract Keyword OR ‘Home Infusion Therapy’ OR ‘Therapy, Home Infusion’ OR ‘Home Infusion Therapies’ OR ‘Infusion Therapies, Home’ OR ‘Therapies, Home Infusion’ OR ‘Infusion Therapy, Home’ in Title Abstract Keyword NOT Neoplasm NOT ‘Glycogen Storage Disease Type II’ NOT ‘Gaucher Disease’ NOT ‘Fabry Disease’ in Title Abstract Keyword	2344
CINAHL	(child OR children OR adolescent OR teen OR teenager OR youth) AND (mucopolysaccharidoses OR mucopolysaccharidosis) AND (“Enzyme Replacement Therapies” OR “Replacement Therapies, Enzyme” OR “Replacement Therapy, Enzyme” OR “Therapies, Enzyme Replacement” OR “Therapy, Enzyme Replacement”) AND (“Therapy, Home Infusion” OR “Home Infusion Therapies” OR “Infusion Therapies, Home” OR “Therapies, Home Infusion” OR “Infusion Therapy, Home”) NOT (Neoplasm NOT “Glycogen Storage Disease Type II” NOT “Gaucher Disease” NOT “Fabry Disease”)	01
Embase	(‘child’/exp OR child OR ‘children’/exp OR children OR ‘adolescent’/exp OR ‘adolescent’) AND ‘mucopolysaccharidosis’ AND (‘enzyme replacement’ OR ‘enzyme replacement therapy’) AND ‘home infusion therapy’ NOT ‘neoplasm’ NOT ‘glycogen storage disease type 2′ NOT ‘gaucher disease’ NOT ‘fabry disease’	04
LILACS	mh:((criança OR adolescente) AND (mucopolissacaridoses) AND (“Terapia de Reposição de Enzimas”) OR (“Terapia por infusões no domicílio”) AND NOT (neoplasia) AND NOT (“Doença de Depósito de Glicogênio Tipo II”) AND NOT (“Doença de gaucher”) AND NOT (“Doença de fabry”))	34
MEDLINE	(((((((“child”[MeSH Terms] OR “child”[All Fields] OR “children”[All Fields] OR “child s”[All Fields] OR “children s”[All Fields] OR “childrens”[All Fields] OR “childs”[All Fields] OR (“child”[MeSH Terms] OR “child”[All Fields] OR “children”[All Fields] OR “child s”[All Fields] OR “children s”[All Fields] OR “childrens”[All Fields] OR “childs”[All Fields]) OR (“adolescences”[All Fields] OR “adolescency”[All Fields] OR “adolescent”[MeSH Terms] OR “adolescent”[All Fields] OR “adolescence”[All Fields] OR “adolescents”[All Fields] OR “adolescent s”[All Fields]) OR “adolescent *”[All Fields] OR “teen *”[All Fields] OR “teenager *”[All Fields] OR “youth *”[All Fields] OR “adolescent * female”[All Fields] OR “adolescent * male”[All Fields]) AND (“mucopolysaccharidose”[All Fields] OR “mucopolysaccharidoses”[MeSH Terms] OR “mucopolysaccharidoses”[All Fields] OR “mucopolysaccharidosis”[All Fields])) OR (“mucopolysaccharidose”[All Fields] OR “mucopolysaccharidoses”[MeSH Terms] OR “mucopolysaccharidoses”[All Fields] OR “mucopolysaccharidosis”[All Fields])) AND “Enzyme Replacement Therapy”[All Fields]) OR “Enzyme Replacement Therapies”[All Fields] OR “replacement therapies enzyme”[All Fields] OR “replacement therapy enzyme”[All Fields] OR “therapies enzyme replacement”[All Fields] OR “therapy enzyme replacement”[All Fields]) AND “Home Infusion Therapy”[All Fields]) OR (“Home Infusion Therapy”[MeSH Terms] OR (“home”[All Fields] AND “infusion”[All Fields] AND “therapy”[All Fields]) OR “Home Infusion Therapy”[All Fields] OR (“therapy”[All Fields] AND “home”[All Fields] AND “infusion”[All Fields])) OR “Home Infusion Therapies”[All Fields] OR (“Home Infusion Therapy”[MeSH Terms] OR (“home”[All Fields] AND “infusion”[All Fields] AND “therapy”[All Fields]) OR “Home Infusion Therapy”[All Fields] OR (“infusion”[All Fields] AND “therapies”[All Fields] AND “home”[All Fields])) OR (“Home Infusion Therapy”[MeSH Terms] OR (“home”[All Fields] AND “infusion”[All Fields] AND “therapy”[All Fields]) OR “Home Infusion Therapy”[All Fields] OR (“therapies”[All Fields] AND “home”[All Fields] AND “infusion”[All Fields])) OR “infusion therapy home”[All Fields]) NOT (“neoplasm”[All Fields]) NOT “Glycogen Storage Disease Type II”[All Fields]) NOT “Gaucher Disease”[All Fields]) NOT “Fabry Disease”[All Fields]	2629
Science Direct	(Child AND Mucopolysaccharidoses AND “Enzyme Replacement Therapy” AND “Home Infusion Therapy” NOT Neoplasm NOT “Glycogen Storage Disease Type II” NOT “Gaucher Disease” NOT “Fabry Disease”)	02
Scopus	(TITLE-ABS-KEY (child OR children OR adolescent OR teen OR teenager OR youth) AND TITLE-ABS-KEY (mucopolysaccharidoses OR mucopolysaccharidosis) AND TITLE-ABS-KEY (“Enzyme Replacement Therapies” OR “Replacement Therapies, Enzyme” OR “Replacement Therapy, Enzyme” OR “Therapies, Enzyme Replacement” OR “Therapy, Enzyme Replacement”) AND TITLE-ABS-KEY (“Therapy, Home Infusion” OR “Home Infusion Therapies” OR “Infusion Therapies, Home” OR “Therapies, Home Infusion” OR “Infusion Therapy, Home”) AND NOT TITLE-ABS-KEY (neoplasm AND NOT “glycogen storage disease type iii” AND NOT “Gaucher Disease” AND NOT “Fabry Disease”))	09
Web of Science	child OR children OR adolescent OR Teen OR Teenager OR Youth (All Fields) AND Mucopolysaccharidoses OR Mucopolysaccharidosis (All Fields) AND ‘Enzyme Replacement Therapies’ OR ‘Replacement Therapies, Enzyme’ OR ‘Replacement Therapy, Enzyme’ OR ‘Therapies, Enzyme Replacement’ OR ‘Therapy, Enzyme Replacement’ (All Fields) AND ‘Therapy, Home Infusion’ OR ‘Home Infusion Therapies’ OR ‘Infusion Therapies, Home’ OR ‘Therapies, Home Infusion’ OR ‘Infusion Therapy, Home’ (All Fields) NOT Neoplasms NOT ‘Glycogen Storage Disease Type II’ NOT ‘Gaucher Disease’ NOT ‘Fabry Disease’ (All Fields)	04
Gray Literature		
Data source	Search strategy	
CAPES Foundation’s Catalog of Theses and Dissertations	Criança OR Adolescente AND Mucopolissacaridoses AND Terapia de Reposição de Enzimas OR Terapia de Reposição Enzimática AND Terapia por Infusões no Domicílio.	00
Google Scholar	Child OR Adolescent AND Mucopolysaccharidoses AND “Enzyme Replacement Therapy” AND “Home Infusion Therapy”	24
NICE	Child OR Adolescent AND Mucopolysaccharidoses AND “Enzyme Replacement Therapy” AND “Home Infusion Therapy”	00
Brazilian Ministry of Health website	Criança OR Adolescente AND Mucopolissacaridoses AND “Terapia de Reposição de Enzimas” AND “Terapia por Infusões no Domicílio”	06
WHO IRIS	Child OR Adolescent AND Mucopolysaccharidoses AND “Enzyme Replacement Therapy” AND “Home Infusion Therapy”	04

**Table 3:** Operationalization of the PAGER framework in the analysis of results.

Patterns	Advances	Gaps	Evidence for Practice	Research Recommendations
Recurring trends in the literature on the topic under investigation	Recent advancements highlighted in the literature	Important issues that have not been addressed or discussed	Relevant and pertinent findings that contribute to supporting evidence-based practice	Recommendations grounded in the identified gaps

## Data Availability

All data used in this study are publicly available, and the respective sources have been properly cited.

## Supplementary Materials

The supporting information can be downloaded at: https://www.mdpi.com/article/10.3390/ijerph22111686/s1, File S1: Prisma-P 2015 checklist. Checklist prism protocol. (DOCX) [15].
